# Safety of Germline Genome Editing for Genetically Related “Future” Children as Perceived by Parents

**DOI:** 10.1089/crispr.2019.0010

**Published:** 2019-12-16

**Authors:** Tetsuya Ishii, Iñigo de Miguel Beriain

**Affiliations:** ^1^ Office of Health and Safety, Hokkaido University, Sapporo, Japan; ^2^ Law and the Human Genome RG, University of the Basque Country UPV/EHU, Faculty of Law, Leioa, Spain; ^3^ IKERBASQUE, Basque Foundation for Science, Bilbao, Spain.

## Abstract

The social acceptability of germline genome editing (GGE) depends on its perceived safety, as well as respect for reproductive autonomy. However, it is doubtful that prospective parents sufficiently understand the risks of GGE. In the future, the use of GGE in specific situations seems plausible, as it offers couples potential means to safeguard genetically related future children from a serious disease and overcome infertility due to a gene mutation. Should GGE fail, however, some couples may be obliged to abort affected fetuses, or give birth to adversely affected children, which would be a tragedy. Some children might develop diseases later in life due to overlooked off-target mutations. Compounding this, some parents are unlikely to inform their offspring about the details of conception, hampering necessary follow-up. Prospective parents, scientists and policy makers should carefully discuss the safety implications of GGE for genetically related future children.

## Introduction

In theory, genetically modifying human germ cells, which include the egg cells, sperm cells, and zygotes (collectively referred to as the germline), can enhance the developmental potential of embryos and result in children with an intended trait. However, due to the inheritance of genetic modification among future generations, germline genetic modification has been tremendously controversial, raising concerns over the safety and welfare of future generations, potential changes to the nature of human reproduction and parent–child relationships, exacerbation of prejudice against people with disabilities, and potential misuse for genetic enhancement.^1^

In the past two decades, some clinics have attempted to use germline genetic modifications primarily to treat intractable infertility.^2,3^ Since 1996, several reproductive techniques involving cytoplasmic or nuclear transfer have been developed to modify the composition of mitochondrial genome (mtDNA) of human eggs or zygotes using donor eggs.^3^ Although some of these cases led to live births, others resulted in miscarriages, chromosomally abnormal pregnancies, and the development of disorders in offspring.^3,4^ Although some countries have prohibited such germline genetic modification, the United Kingdom became the first nation explicitly to permit two types of cytoplasmic replacement using nuclear transfer to exclude most mutated mitochondria in the eggs or zygotes (mitochondrial donation) in 2015 in order to allow prospective parents to have genetically related children free from serious mitochondrial disease.^5^

Recently, genetic modification technologies using programmable bacterial nucleases (DNA-cutting enzymes), collectively called “genome editing,” have spread worldwide as efficient, versatile, and cheap genetic engineering tools. One of them, CRISPR-Cas9, uses nucleases and programmable guide RNA molecules to modify specific genes in the nuclear and mitochondrial genomes of various species.^6^ Since 2015, basic research on human germline genome editing (GGE) has proceeded toward clinical applications to prevent genetic disease prenatally.^7–9^ In contrast to germline genetic modifications through cytoplasmic or nuclear transfer, GGE technically does not depend on gamete donation from third parties. It requires only the introduction of programmed nucleases into the germline. Therefore, it is likely that more prospective parents will consider GGE more favorably. Indeed, a recent survey found that approximately 60% of respondents accept GGE for medical purposes, whereas only 27% accept it for nonmedical purposes.^10^

Nonetheless, reports on basic research into GGE technology have still stirred a fierce global debate. In recent years, international societies and communities have issued more than 60 ethics statements regarding GGE.^11^ Notably, a 2017 report by the U.S. National Academies of Sciences, Engineering, and Medicine (NASEM) concluded that GGE trials might be permitted only after further preclinical research clarifies the potential “risks and benefits,” and only for “compelling medical reasons … [in the] … absence of reasonable alternatives.”^12^ Additionally, a 2018 report by the Nuffield Council on Bioethics (NCB) in the United Kingdom concluded that GGE could be acceptable if it is intended to secure, and be consistent with, the “welfare …[ of the] … future person” and should not increase disadvantage, discrimination, or division in society.^13^

At the end of 2018, a Chinese researcher, who asserted that his research conformed with the guidelines of the NASEM report, claimed that twin girls had been born safely via GGE using CRISPR-Cas9.^14^ Subsequently, a Chinese regional government confirmed their births but found serious compliance violations.^15^ In this use of CRISPR-Cas9, the researcher attempted to introduce a CCR5 gene deletion that confers resistance to human immunodeficiency virus (HIV) infection (naturally found in approximately 10% of Northern European populations). Importantly, the father of the twins was HIV-positive, and both parents considered that providing HIV resistance for their future children would enhance the welfare of their offspring. However, in this and other cases, GGE might unintentionally cause off-target mutations in genes that are important for health, which can potentially affect the resultant embryos, fetuses, and children systemically, infringing on their human rights.

Therefore, we must be aware that a world where GGE is available either legally or illicitly is imminent. Before prospective parents with autonomy widely pursue experimental GGE, it is imperative that we consider the safety of GGE and its social implications. To facilitate much-needed discussion, the present article will examine the safety of GGE for medical purposes.

## Differences Between Somatic Editing and Germline Editing

Previously, in two trials of conventional somatic gene therapy for a severe combined immunodeficiency (SCID), 5/20 subjects developed leukemia several years after the administration of CD34+ progenitor cells in which a retroviral vector encoding *IL2RG* was introduced.^16,17^ The side effect of leukemia was due to the activation of protooncogenes caused by the genomic insertion of retroviral vectors in an unintended manner. Physicians treated the five affected subjects, and while four recovered, one ultimately died. However, the safety of viral vectors has recently been improved.^18^ Currently, clinical trials using somatic cell genome editing (SGE) are ongoing to develop novel therapies for patients with cancers and genetic diseases.^6^ In SGE, programmed nucleases are introduced into somatic cells, and then a target DNA sequence is efficiently cut and modified for a therapeutic effect. However, there remains a risk of unintentional large-scale rearrangements or small insertion and deletion mutations at off-target sites,^19^ which could result in serious side effects, including tumor formation through the activation of protooncogenes or disruption of tumor suppressor genes.^20^ Despite the different mechanisms between SGE and more traditional gene therapy, such unintended genetic modifications are irretrievable and persistent in some cells of subjects, which drastically differentiates SGE and gene therapy from chemical agents that are soon metabolized and excreted from the body.^20^ Before obtaining consent from volunteers, the potential risks and burdens, as well as the benefits of SGE, must be carefully explained to them.

The use of germline genetic modification in the context of contemporary reproductive medicine introduces more complex ethical issues into the debate. In this case, the individuals providing consent will be prospective parents. However, the actual subjects being directly affected will be eggs or sperm cells, embryos, and ultimately children via reproduction. Of course, unborn children cannot be informed of the risks and cannot give consent. Again, we consider the case of germline genetic modifications through cytoplasmic or nuclear transfer, including mitochondrial donation. These processes involve the transfer of mitochondria (containing their mitochondrial genome) from eggs donated from a third party, and thus are controversial due to their involvement of a third “parent” in the *in vitro* fertilization (IVF) process.^21,22^

The direct use of donor eggs can help prospective parents have children, but at the same time, egg donation has raised ethical issues concerning female exploitation and the commodification of eggs in addition to potential harms to female donors.^23^ Moreover, the intended mother is not genetically related to the donor-conceived children, which can also lead to concerns, such as emotional conflict over whether to disclose the fact of donor conception to the children,^24^ and problems of resemblance due to a lack of physical similarity between the mother and such children.^25^ Mitochondrial donation could alleviate these concerns by helping prospective mothers who consent to it to have genetically related children (genetically related children free from mitochondrial disease when the prospective mothers have a pathogenic mtDNA).

The fact that GGE does not technically require gamete donation will make it more appealing to prospective parents, including parents who seek genetically related children free from disease, as well as parents wishing to overcome infertility due to a gene mutation.^26,27^ However, there is no medicine without risks. In the above-mentioned Chinese GGE case, several embryos that underwent genome editing were tested for the presence of intended and unintended genetic modifications. Then, selected embryos were transferred to a prospective mother. In this context, we note that genetic testing of modified embryos may overlook unintended, small genetic modifications that result from the use of insufficiently programmed nucleases.^26^ Although the author of the Chinese report claimed that the GGE process ended in healthy births, it is unlikely that all GGE cases will proceed without risks to the resultant embryos, fetuses, and children. As such, no ethics committees are likely to approve large-scale GGE studies enrolling hundreds or thousands of couples, even though some authors have asserted that large studies are needed.^28^ Indeed, GGE studies are likely to be limited to small, open-labeled, uncontrolled case series, if performed at all. Although GGE might attract many prospective parents who have reproductive autonomy, it is likely to remain an experimental intervention to human reproduction for a long time due to the limitations of clinical study.

## Moral Status of Human Embryos in GGE

It is worth exploring divergent views on prenatal life in prospective parents who consider GGE. In regard to the moral considerations regarding the human embryo and fetus, we can describe the three main outlooks as the “all,” “none,” and “gradualist” positions.^29^

### The “All” position

Those adopting the “all” position hold that human embryos already possess full human status. For them, germline genetic modifications, including GGE, are in themselves acceptable because these unborn “humans” who are suffering from a genetic problem deserve medical attention.^30^ At present, however, those holding the “all” position have come to regard such experimental interventions as unethical, since a relevant addendum to their position is that there be no risk of adverse events, no use of reproductive techniques, and few or no wasted embryos (humans).^31^ Again, however, they would essentially accept the use of GGE for ensuring the welfare of unborn “humans” if GGE was perceived as safe, if it was not considered reproductive medicine, and if it would contribute to reduce the number of created embryos and, consequently, the number of spare embryos.

### The “None” position

Conversely, the “none” position asserts that human embryos or fetuses have no moral status and therefore deserve no special moral concern before childbirth. As such, those holding the “none” position largely accept germline genetic modifications that can help them to have genetically related children.

### The “Gradualist” position

The “gradualist” position regards human embryos as potential human beings, but not actual humans until birth. It also considers that human embryos possess a special status that deserves a certain degree of respect, which increases along with their development. Similarly to the “none” position, the “gradualist” position may accept germline genetic modifications for both clinical and research purposes. However, prospective parents are likely to encounter dilemmas when embryos and fetuses implanted in the mother are adversely affected. While they may accept that such embryos and fetuses are morally different from actual human beings, they may still feel that these entities should be treated with a higher degree of respect than genome-edited embryos that have yet to be implanted. In so doing, it seems unclear whether such affected implanted embryos and fetuses should be medically treated, as they were in the SCID gene therapy trial.

## Scenarios After GGE and Psychological Aspects of Parents

To consider scenarios that might arise after GGE, the first human germline genetic modification is revisited below. From 1996 to around 2002, an American clinic performed a small study to test a germline mitochondrial modification technique for intractable female infertility, wherein the cytoplasm (containing mitochondria) from a donor egg is transferred into a patient's eggs (cytoplasmic transfer).^4^ The study helped 13 couples have 17 genetically related children, but also resulted in a miscarriage, probably due to a chromosomal abnormality (Turner syndrome), and one selective fetal reduction from a twin pregnancy (Turner syndrome). While the pregnant woman might have been able to support both twins, she selectively aborted the affected fetus, where the mother's reproductive autonomy is generally permitted to take precedence over the life of the embryo or fetus.^32^ Of note, it is virtually impossible to treat affected fetuses fundamentally in cases where many or all of the cells are genetically abnormal. Likewise, in GGE, while some parents may have genetically related children, others may choose to abort adversely affected fetuses.

Pregnant women frequently opt for abortion when prenatal testing reveals a genetic abnormality in their fetus.^33^ In terms of abortion in cases of germline genetic modification, further exploration of the psychological aspects of the parents is needed due to the potential risks to unborn children of parents who adopt the “all position” or to potential children for those who take the “gradualist position.” In contrast to the cytoplasmic transfer study, the Chinese GGE case and most GGE basic research intend to prevent the onset of a disease in offspring prenatally, despite etiological differences. If prospective parents who have consented to GGE for that medical purpose, they are not harboring a vague desire for healthy children but clearly wish to safeguard genetically related children against a specific disease. Nonetheless, if issues arise with the genetic intervention, they may be forced to choose whether to abort the affected fetuses—namely, whether to “kill unborn or potential children.” Such conflict would likely cause substantial grief to such couples.

## Safety of GGE for Future Children

From a broader perspective, abortion can play a large role in integrating GGE into society. Indeed, the U.S. cytoplasmic transfer study, in which one affected fetus was aborted, helped 13 infertile couples to have genetically related children. However, 1 of the 17 children was subsequently diagnosed with a borderline pervasive developmental disorder, resulting in a regulatory intervention by the U.S. Food and Drug Administration.^34^ For genome editing, a target sequence-binding molecule is designed primarily using a reference genome. However, the human genome differs slightly among individuals,^35^ which may mislead scientists programming nucleases and result in unintended genetic modifications. In addition, there is no perfect prenatal testing. Should GGE become widespread, these limitations of genome editing and prenatal testing may lead to the birth of some adversely affected children or the later development of disease in some of these individuals after they grow. How then should the safety of experimental GGE be considered?

Once again, we might use as a paradigmatic example the discussions surrounding mitochondrial donation in the United Kingdom. In this case, a regulator's panel concluded in 2013 that “evidence available at that time did not suggest that the techniques are unsafe,”^36^ which in part led to the legalization. This suggests that possible points to consider regarding the safety of GGE include the presumed probability and seriousness of adverse events at birth and/or at some future points after preclinical research defined the degree of safety. Is it acceptable so long as the probability of adverse events after GGE is far less than one adverse event in 17 children in the case of cytoplasmic transfer? For some, such a lower probability may be acceptable, as most couples have genetically related healthy children. However, in addition to this probability, the seriousness of conditions of affected children should also be addressed.

On closer examination, unsafe mitochondrial donation has two different implications. First, the germline mitochondrial modification may fail to prevent the inheritance of serious mitochondrial disease to offspring. This failure would then result in the birth of children affected by a serious mitochondrial disease. Second, mitochondrial donation may affect the resultant children in an unexpected manner, imposing serious conditions other than mitochondrial disease upon them. Such conceivable risks to future children eventually did not halt the legalization of this procedure in the United Kingdom because those risks were perceived as acceptably low. Likewise, when preclinical research further advances, some countries could justify the clinical use of GGE for medical purposes, probably including the prenatal prevention of serious disease.

One might view the Chinese researcher's claim that twins were born safely via GGE as similar to the first successful birth using IVF in 1978, which led to wide use of technique worldwide. However, small off-target mutations might cause health complications in the children as they grow. It is therefore crucial to conduct long-term follow-up of children born via GGE.^37^ Regarding mitochondrial donation, the regulator in the United Kingdom only requires that reproductive scientists prepare a long-term follow-up plan,^38^ suggesting that the long-term follow-up after GGE will also be left up to scientists and parents. However, not all the children are likely to undergo the necessary follow-up. This is not simply because parents who consented to follow-up may later withdraw that consent. Of note, in the survey results of families who joined the cytoplasmic transfer study, only 1 out of 13 couples disclosed the use of the germline genetic modification to their children, which appears to be lower than the disclosure rate after the direct reproductive use of donor eggs.^4^ This low disclosure rate is probably associated with the involvement of “experimental” infusion of mitochondria derived from donor eggs, which makes parents hesitate to disclose the fact of conception to their children. In such a situation, children who are not informed of the fact that they were born using experimental reproductive medicine may sometimes decline important hospital visits simply because they do not understand that their genome was edited, and that medical examinations are important to offset the potential risks associated with genome editing. Thus, their health may be threatened in the future.

The regulator in the United Kingdom requires reproductive scientists to report childbirth with mitochondrial disease, birth defects, genetic abnormalities, or other adverse events after mitochondrial donation was performed.^38^ Aside from issues with long-term safety, consider a situation in which the use of GGE for preventing serious disease in future children unexpectedly results in the birth of one child afflicted with another serious illness. It may be impossible to treat such a child fundamentally, as unsafe genome editing has unintentionally modified many or all of their cells. This would be a tragic event for all parents, regardless of their views on prenatal life, and some parents might even bring a wrongful birth lawsuit against reproductive scientists.^39^ However, this lawsuit would likely become a protracted court case, as it would be unclear whether adverse events occurred due to the side effects of GGE or to the genomic instability frequently observed in the early embryos.^40^ Furthermore, unsafe GGE may later result in family discord. In some countries, the affected child could bring a wrongful life lawsuit against their parents in addition to reproductive scientists, claiming that she or he should not have been born.^39^ However, such actions are unlikely to be taken by the child because the parents refrain from disclosing the fact of their conception involving an experimental genetic intervention. The socially permissive politics that gave rise to the legality of wrongful birth lawsuits could backfire and diminish the rights of individuals whose parents attempt to guard preemptively against wrongful life lawsuits by withholding the facts of their conception.

Regarding conventional gene therapy, nearly 3,000 trials have been performed worldwide, with a dozen or more approved therapies.^26^ Several SGE trials are ongoing at present. With prior review and patient consent, such clinical efforts are worthwhile because the development of SGE as well as gene therapy may satisfy the needs of current disease sufferers. This is in contrast to germline genetic modifications. Those who consent to such germline interventions are prospective parents. Current research reports suggest that most of the subjects would be preimplantation embryos with or without genetic defects that are unborn children for the “all” position but are morally different from existing humans to those adopting the “gradualist” or “none” position. For prospective parents with reproductive autonomy, GGE can be viewed as wish-fulfilling medicine.^41^ Namely, GGE can help such parents create genetically related children with or without a specific trait. In contrast, some argue that germline genetic modification has few compelling needs and little social value.^21,22^ In addition, unintended off-target mutations may adversely affect germline cells, potentially ruining the welfare of the resultant children, which conflicts with the ideals laid out in the NCB report. If countries judge that the potential harms to future children as well as the relative paucity of compelling needs or social value outweigh parental wishes for having genetically related children, they will maintain or prohibit GGE. On the other hand, in the United Kingdom, mitochondrial donation is legal for prospective parents pursuing genetically related healthy children. Therefore, the acceptability of GGE largely depends on its safety, as well as the degree of respect for parental reproductive autonomy. Is the risk of unintended victims of GGE an acceptable risk for such parents? At present, it is doubtful whether prospective parents sufficiently perceive the safety implications of GGE.

## Conclusion

Given that GGE is more likely to spread than older germline genetic modifications, and that GGE carries a real risk of adversely affecting children with human rights in an irretrievable manner, it is vital for prospective parents with reproductive autonomy, as well as scientists and policy makers, to perceive the safety implications of experimental GGE for the genetically related future children. It also important to open a social debate on the necessity of disclosing the use of GGE to the Chinese twins and individuals whose birth involved the technique if we wish to guarantee their fundamental rights.
